# A cost effective communication model for requirements elicitation in global software development

**DOI:** 10.1038/s41598-023-45415-6

**Published:** 2023-10-31

**Authors:** Muhammad Aqib Rauf, Sarfraz Bibi, Sadia Ali, Tahani AlSaedi, Saif Ur Rehman, Khalid Mahmood, Mahwish Kundi

**Affiliations:** 1 University Institute of Information Technology, Pir Mehr Ali Shah Arid Agriculture University, Rawalpindi, Pakistan; 2 Applied College, Taibah University, 42353 Madina, Saudi Arabia; 3 Institute of Computing and Information Technology, Gomal University, D.I. Khan, 29220 Pakistan; 4 Department of Computer Science, Abdul Wali Khan University Mardan, Mardan, Pakistan

**Keywords:** Engineering, Mathematics and computing

## Abstract

Requirement elicitation stands as a pivotal activity within requirement engineering, gaining even greater significance in the context of global software development. Effective communication among stakeholders assumes paramount importance in this arena. Factors such as time zone disparities, cultural variations, and language differences exert a formidable impact on communication within the sphere of global software development. These dynamics inevitably impinge upon timely coordination, potentially compromising the software's quality. In response, researchers have proffered communication models tailored for requirement elicitation within the ambit of global software development. The purpose of this study is to conduct an in-depth critical review of existing communication models for demand elicitation in global software development. Through this comprehensive review, we aim to discern prevailing publication trends, provide an introductory overview, and illuminate the strengths and limitations inherent in the existing communication models. By identifying these limitations, we seek to advance a novel, low-cost communication approach designed primarily for demand elicitation in global software development. To culminate our endeavor, we will undertake a case study-based experiment, meticulously designed to assess the efficacy and practical utility of the proposed techniques.

## Introduction

Requirement engineering (RE) is a multi-stage, disciplined technique for identifying and implementing software requirements^1^. This multi-staged strategy includes many critical operations, such as requirement elicitation, the creation of software requirement specification papers, the verification and validation of requirements, and the management of requirement change^2^. The most important activity, which influences the quality of the resulting software, is requirement elicitation.

Proper requirement elicitation necessitates timely communication and collaboration among all parties. Communication and coordination become even more crucial when players are dispersed around the world^2^. Eliciting needs becomes more challenging when stakeholders are spread throughout the globe due to time zone, cultural, and linguistic variations^3^.

Despite the fact that English is considered a global communication standard, cultural differences continue to generate communication issues. Global software development is a new branch of software engineering that aims to optimize resources, increase market share, and reduce costs by distributing software globally^4^.

However, in addition to these advantages, global software may run into problems with knowledge management, group awareness, communication, collaboration, and project management. Global software development communication is based on groupware technologies such as email, video conferencing, and instant messages. Long response times, poor communication, a lack of face-to-face involvement, and misunderstandings can all lead to annoyance and a loss of motivation and interest^5^. Participation is often hampered by cultural and time disparities, leading to a lack of trust and misconceptions when resolving problems. To get the full benefits of global software development, emphasize strong communication, particularly during demand elicitation. A communication framework for eliciting needs for global software development has already been presented by researchers. Our research will begin with a detailed examination of existing communication approaches for eliciting demand in global software development^6^. Conducting this review holds the promise of illuminating the limitations inherent in prevailing approaches these limitations are task allocation, irrelevant requirement selection and irrelevant requirement identification. Armed with insights gleaned from these identified constraints, we endeavor to fashion a novel and cost-effective communication model tailored specifically for requirement elicitation within the domain of global software development. Addressing various challenges, we intend to mitigate issues such as the absence of comprehensive stakeholder training, the lack of virtual assistants for stakeholders, inadvertent selection of irrelevant requirements, inadequate process or task allocation mechanisms, and a dearth of checks on duplicated requirements. By harnessing the lessons derived from these limitations, our proposed communication model aims to foster enhanced collaboration, streamline processes, and ensure the holistic selection of pertinent requirements. With this approach, we aspire to elevate the quality of requirement elicitation within the expansive realm of global software development.

## Related work

This section provides an overview of the communication techniques available for eliciting requirements in global software development. The primary goal is to present a current overview, contribution, suggested methodologies, and limitations of existing communication systems proposed for global software development. The authors conducted an empirical investigation to identify issues in global software development. The communication, coordination, and cultural issues that exist in current approaches were highlighted in this empirical investigation. Based on these limits, a new paradigm for addressing these difficulties has been proposed^7^. The proposed paradigm prioritizes communication and cultural diversity as two important concerns in global software development. Some authors presented a survey to identify the root causes of requirements engineers' and customers' communication issues. A poll was conducted for this purpose, with employees from one software development company participating. The data suggests that communication problems arise when stakeholders are not involved. Furthermore, miscommunication between the developer and the consumer causes problems with communication, coordination, and collaboration because they cannot easily understand each other's thinking^5^. A framework for better communication while collecting requirements for global software development. Case-based reasoning is used in global software development to establish the best elicitation technique for demand elicitation. The proposed framework is broken into two sections. The first phase addressed cultural, language, and time zone difficulties. In the second step, the requirement elicitation approach was used, and requirements were acquired from stakeholders^8^. Good specification debate and negotiation is a significant hurdle to overcome in GSD, but it is impossible to hold good requirements discussions due to the aforementioned issues, such as time gaps, language limits, and cultural differences^9^. Without trust, there can be no partnership or teamwork, and without cooperation and trust, development can only happen by chance. Implementing requirements correctly is a difficult task, as is obtaining user demands for development. A suitable and proper level of communication among stakeholders is required, with the main role being to collect the requirements. Investigate the role of communication between various sites. The significance of efficient communication among stakeholders in requirement collection was emphasized. The goal is to supply all of the solutions and practices required for effective worldwide software development communication^10^. Requirement elicitation is one of the most important and visible tasks in software development. The project fails if it does not function properly. The primary reason for failure is insufficient requirement gathering. Their purpose is to map out the technical responsibilities of RE engineers. Work on the two-requirement elicitation technique, which includes ethnography and interviews. It should be emphasized that one requirement engineer cannot manage both requirement methodologies and different elicitation techniques simultaneously. One personality is said to be incapable of managing both the chosen technique and the personality of the other^11^. This study stressed human capabilities in connection to engineering job requirements. Obtaining product requirements is the most critical job during software development. During requirement collection, we used a framework to improve communication and coordination difficulties among stakeholders, and we chose the best and most relevant ways for them to elicit the demand, which was case-based reasoning in a GSD context. This study employed a case study in their framework to build communication skills, determine the best elicitation technique, and give communication advice to the requirement engineer. The problem is that there is no way to accommodate redundant requirements^12^. According to studies, requirement implementation is a challenging task in software engineering. In global software development (GSD), gathering user needs is getting increasingly complex. Effective client-vendor communication is important to the GSD's capacity to successfully gather requirements. It is vital to research how the importance of efficient communication changes between continents, historical events, and organizational sizes. To attain aims and objectives, a systematic literature review (SLR) is used^13^. Stakeholder satisfaction is crucial in dispersed and global software development, and stakeholder communication raises a number of difficulties. As a result of this research, a framework for optimizing the management process and component processes in remote development was proposed^14^. To decrease uncertainty and incompleteness among stakeholders, create a sentimental aspect-based analysis. Use a case-based reasoning method and a tree-based taxonomy of needs to encourage stakeholder involvement. As a result, product quality improves with less redundancy and irrelevancy among stakeholders, aspects, and priorities.

## Proposed approach

As a previous outcome, we identified and discovered that there is now a realistic and perceived demand for the development and analysis of new and more efficient, cost-effective communication for requirements elicitation in global software development, thereby reducing the barriers commonly encountered in global software development and providing the truthfully and very impressive results of a cost-effective communication mode. In this chapter, we will provide a brief overview of the cost-effective communication model approach, which is intended to be a cost-effective technique for making the requirement-gathering process easy and simple, making it ideal for use in a global software development environment. We will present the research approach used to support this study activity, which is based on guided collaborative workshops and requirement elicitation software development. As a result, action research is being used to do this research. Action research is a method of systematic and collaborative inquiry that entails identifying a specific problem or challenge in a real-world environment and then engaging in a cycle of reflective and iterative procedures to collect data, analyze results, and implement changes or solutions. We attentively discovered and thoroughly investigated each distinct challenge offered during our investigation. As a result, we focused our efforts on developing effective and practical solutions to each new difficulty that arose. A robust validation process involving comprehensive testing, analysis, and assessment was carried out to confirm the viability and efficacy of these solutions. This section also contains a full review of all of the steps taken to complete this research. We start by going over previous work on the topic of cost-effective communication in requirement elicitation. The purpose of this research is to present a way for executing process and cost communication models in global software development for requirements elicitation.

### Description of proposed framework

Effective communication for requirements elicitation prior to project start is crucial for project rejection or acceptance. In the realm of global software development, several methodologies and frameworks are utilized for demand elicitation. Stakeholders encounter a variety of challenges and constraints, and some of them specify success criteria. Because of the benefits of decreasing development costs, the global software development trend is empowering enterprises. The necessity for the proposed solution was formed by the scenario of many study articles and their literature reviews, hypotheses, case studies, surveys, and other approaches. To overcome the constraints of global software development that face stakeholder groups, we created a technique termed a cost-effective communication model for requirements elicitation in GSD.

**Figure 1:**
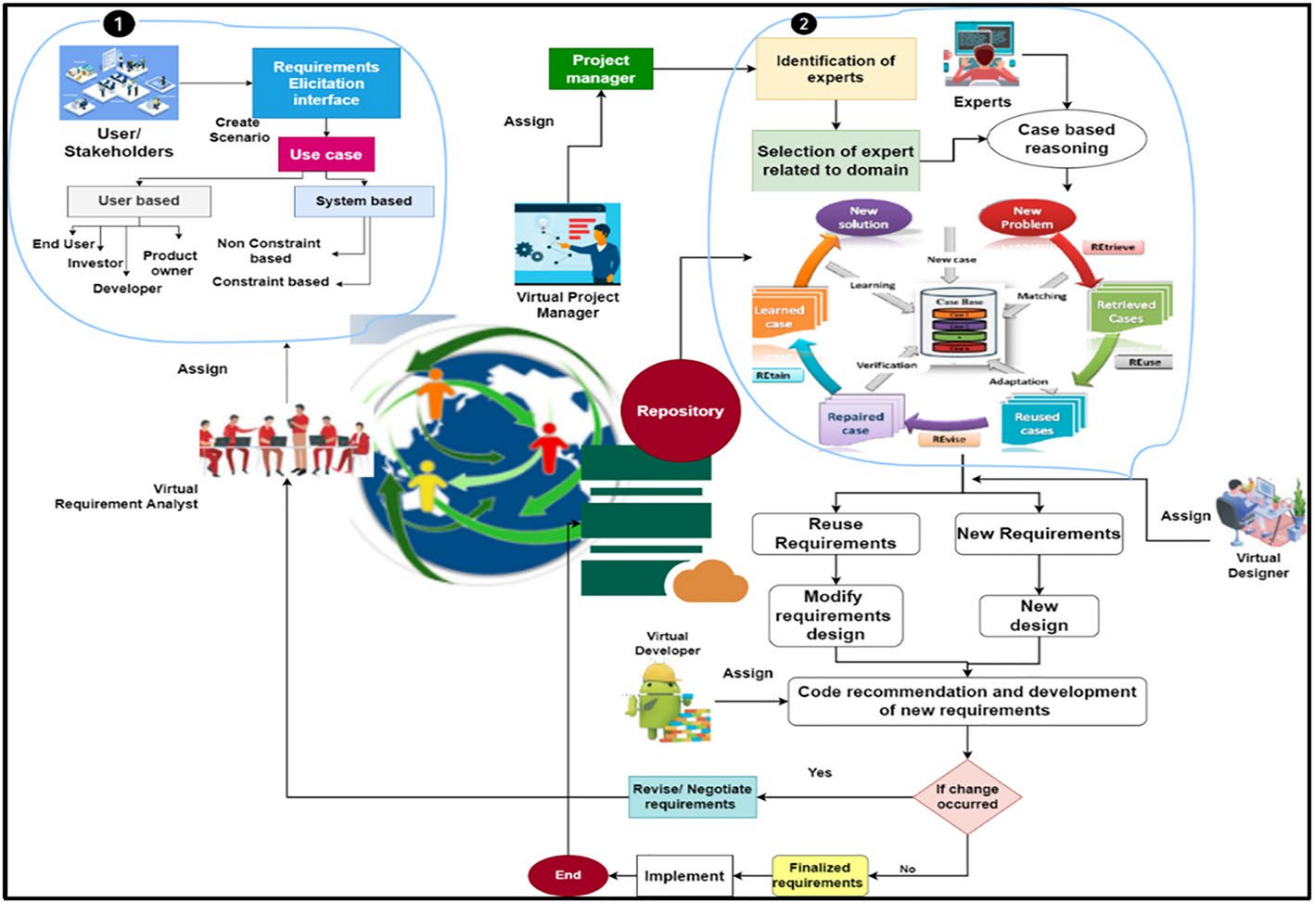
Proposed approach.

**Figure 2:**
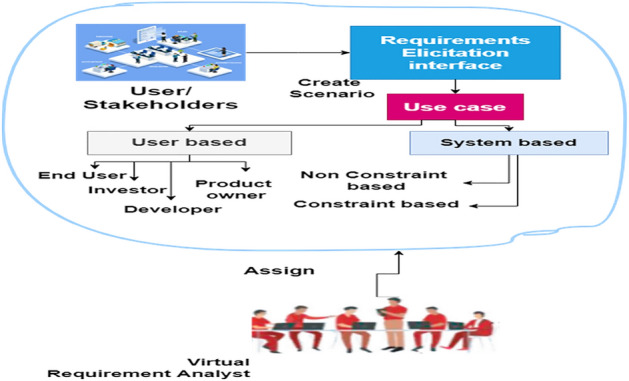
Requirements elicitation.

**Figure 3:**
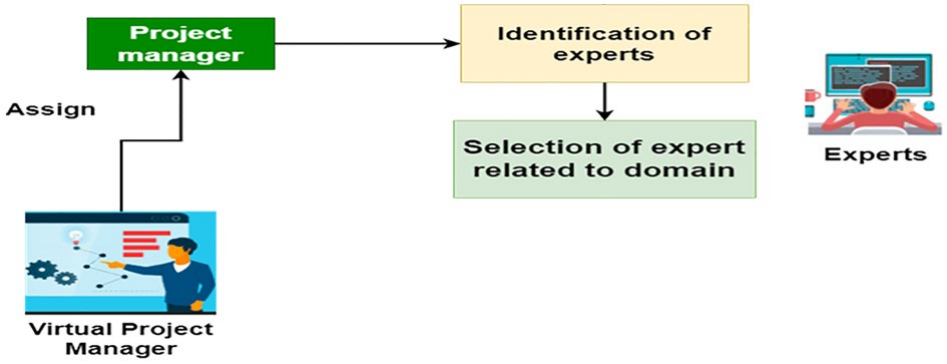
Role of project manager.

**Figure 4:**
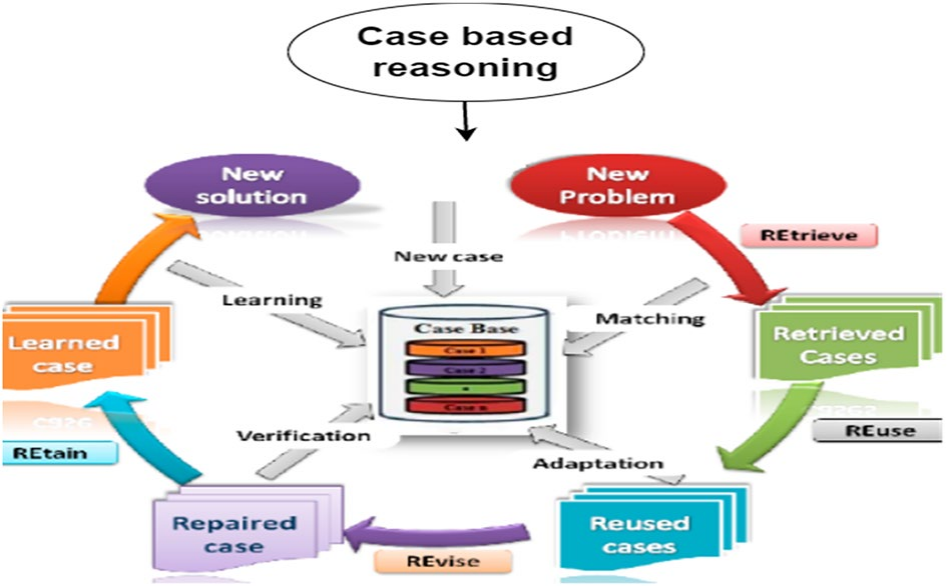
Role of case based reasoning.

**Figure 5:**
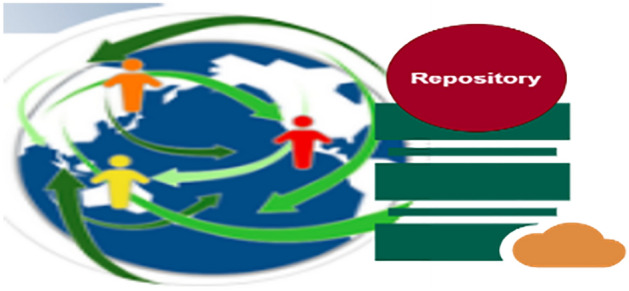
Role of repository.

**Figure 6:**
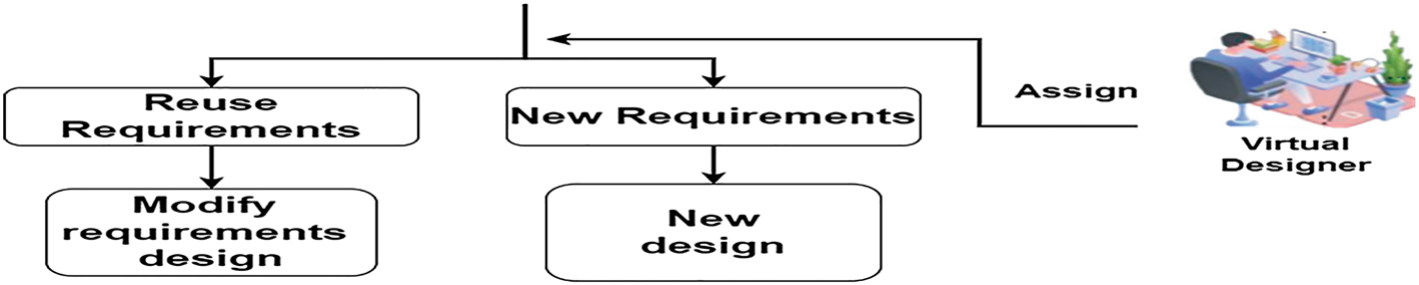
Role of designer.

**Figure 7:**
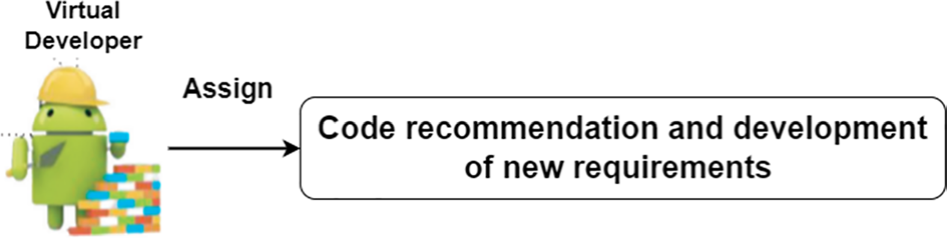
Role of developer.

**Figure 8:**
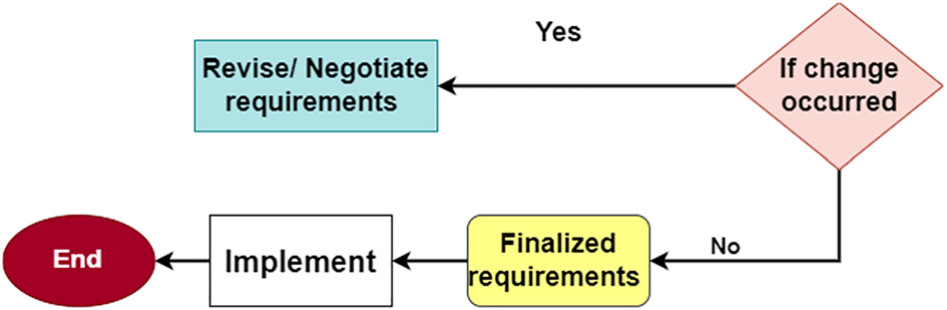
Finalized the requirements.

We defined the summary of our framework in this section by using conceptual components or framework modules to explain the various process activities based on the chain of work. Based on the process, the suggested framework is divided into various stages, and each phase's actions are explained for cost-effective communication and demand elicitation, with their activity depicted in Fig. 1. All of the images Figs. 1, 2, 3, 4, 5, 6, 7 and 8 have been generated using this software draw.io and are accessible through the same link (https://app.diagrams.net/), providing a seamless and convenient way to access and share visual content effortlessly. This cohesive approach not only streamlines the process of viewing and managing images but also ensures consistency and accessibility for all users, enhancing collaboration and communication within the digital environment.

### Requirements elicitation interface

To begin, we provide a help box from which you may select or recommend a requirements analyst who is available 24 h a day, seven days a week. All team members were effectively linked on the crowdsourcing platform as a result of this. As shown in Fig. 2, the tool changes or randomly offers the parameter that will be employed or is randomly offered by the tool whose team members are suitable for this activity. We have a single system or application interface via which users or stakeholders can engage with or discuss the project, and we then design an interface scenario for requirements elicitation. User/ Stakeholders, Scenario of interview, Use case, User based, End user, Investor, Developer, Product owner, System Based, Constraint based, Non constraint based. The image was created using draw.io, highlighting the platform's versatile diagramming features.

### Project manager

In our system, we appoint a virtual project manager to oversee project management throughout this vital phase. Project managers also interact with users or clients with the help of experts, first attempting to identify experts and then selecting experts who are suitable and related to their domain because some may be developers, finance professionals, or other user types, as well as checking team members and their geographical location, verifying project documentation requirements, and determining which developer's or stakeholder's members are suitable for this rejection. The header in the second slot contains already assigned project information or a mockup of past projects that have already been worked on, implying that there are numerous project prototypes available there, as seen in Fig. 3. The image was created using draw.io, highlighting the platform's versatile diagramming features.

### Case based reasoning

Case-based reasoning is a strategy and experience-based method for tackling new problems by using previously successful solutions to similar obstacles and identifying how to approach these difficulties and challenges. The use of CBR is highly significant in our research and a novel idea that includes a variety of cases: first a new problem occurs, then the case is retrieved, reused, repaired, and learned, and finally, a new solution is learned and implemented for future use. Employing the technique of case-based reasoning, our approach unfolds across a structured framework encompassing six distinct stages. Our work diligently progresses through each of these stages, laying a robust foundation for subsequent processes. Notably, Techflow International Company embraced and implemented this model, leading to tangible and commendable outcomes. The application of our model by Techflow International resulted in notable enhancements in both results and accuracy, underscoring the effectiveness and practicality of our chosen approach. CBR has several stages, cases, and sorts of issues that it recovers and reuses, as indicated in Fig. 4. Utilizing draw.io, the image was crafted, emphasizing the platform's versatile diagramming tools.

Process of case base reasoning involves:

1. *Case retrieval* Identifying relevant cases from the existing knowledge base that are similar to the current problem or situation.
2. *Case reuse* Applying solutions or strategies from retrieved cases to the current problem, making necessary adaptations if required.
3. *Case revision* Modifying or updating the retrieved solutions based on the specific context or changes in the problem domain.
4. *Case retention* Storing the adapted solutions and the experience gained from the current problem-solving process in the knowledge base for future use.

This iterative process allows for learning from past experiences and applying them to new scenarios, enhancing decision-making and problem-solving in various domains.

### Repository

A repository is a location, database, or box where needs are kept. All actions and procedures, as well as CBR cases, are linked to the repository. All requirements can be viewed and reused by requirement analyzers as well as other developers and designers. Before adding the extra criteria, we can check the repository to determine if the mockup shown in Fig. 5 is still available. Utilizing draw.io, the image was crafted, emphasizing the platform's versatile diagramming tools.

### Virtual designer

We assign the virtual designer to examine the requirements in the two stages of reuse and new needs using case-based reasoning. The designer builds a design using specifications that he may adjust. If the specifications change, he creates a new design and description sample, as illustrated in Fig. 6. Crafting the image was made possible through draw.io, underscoring the platform's adaptable diagramming tools.

### Virtual developer

After the designer has completed the design of new needs or made changes to existing requirements, assign a virtual developer. The designer and developer exchange messages to discuss the requirements indicated in Fig. 7. Crafting the image was made possible through draw.io, underscoring the platform's adaptable diagramming tools. We assign the code recommendation and create new requirements when a requirement changes.

### Finalized of requirements

This process has two stages in which modifications can occur. If adjustments are needed, amend or negotiate the requirements after hearing from the other members. If no adjustments are required, the requirements are complete, and the implementation and testing, as shown in Fig. 8, are followed. The image came to life with the assistance of draw.io, underscoring the flexibility of its diagramming tools.

## Algorithm of framework

The algorithm step is divided into three sections. The first step is requirement elicitation, which is the process of obtaining needs and is illustrated in Table 1.

**Table 1:**
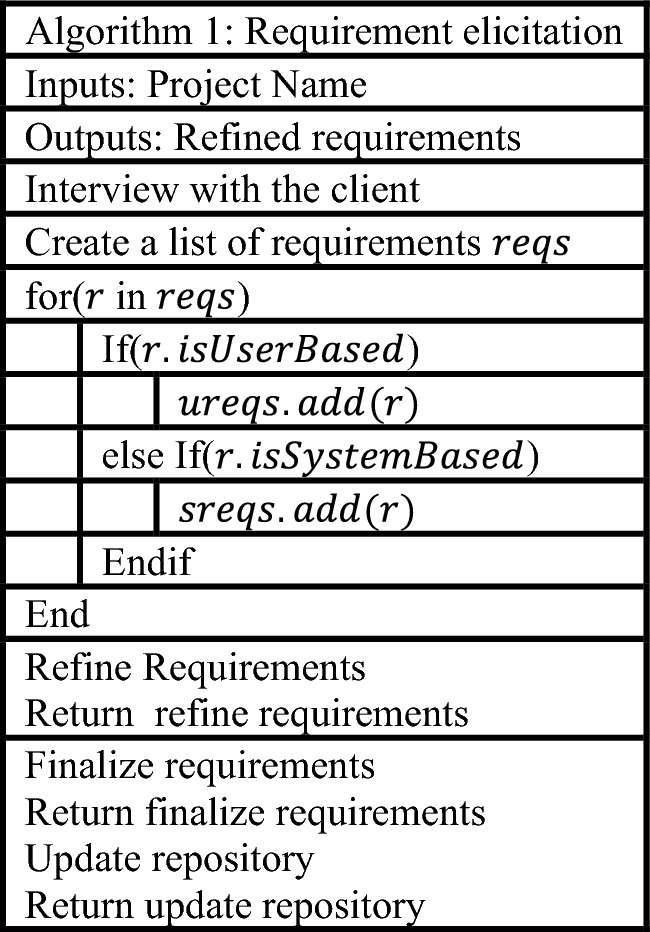
Requirements elicitation.

### Requirement gathering

In the second phase, the project manager distributes team members based on the project's domain which shown in Table 2.

**Table 2:**
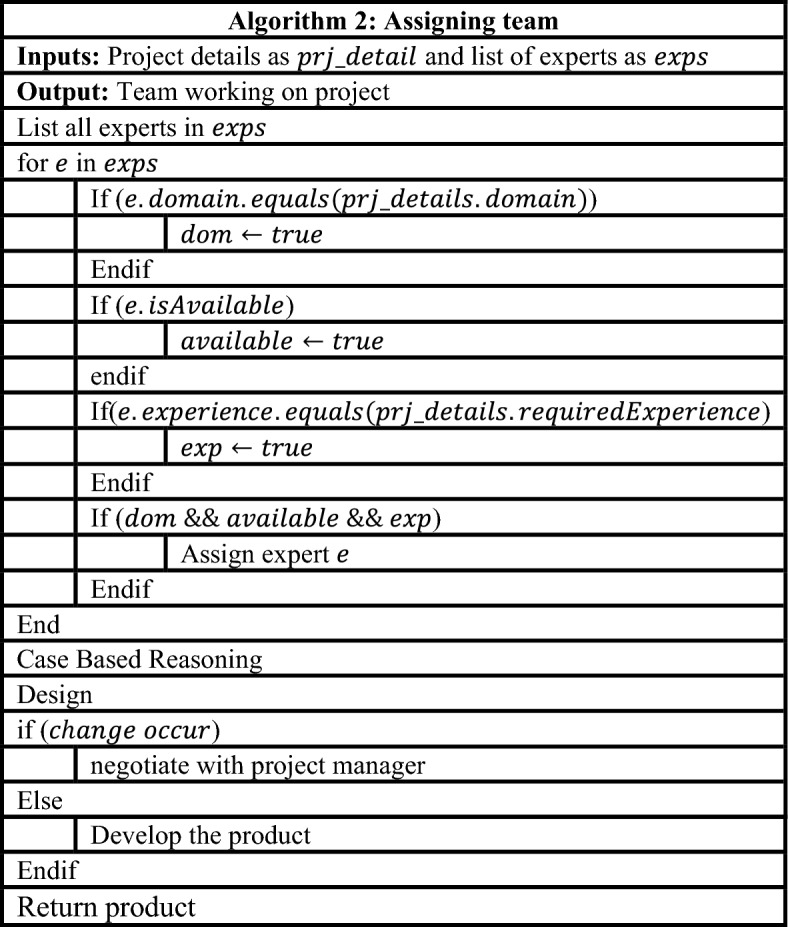
Assigning team.

In the third phase, apply case-based reasoning developing new requirements or reused requirements for future purposes which shown in Table 3.

**Table 3:**
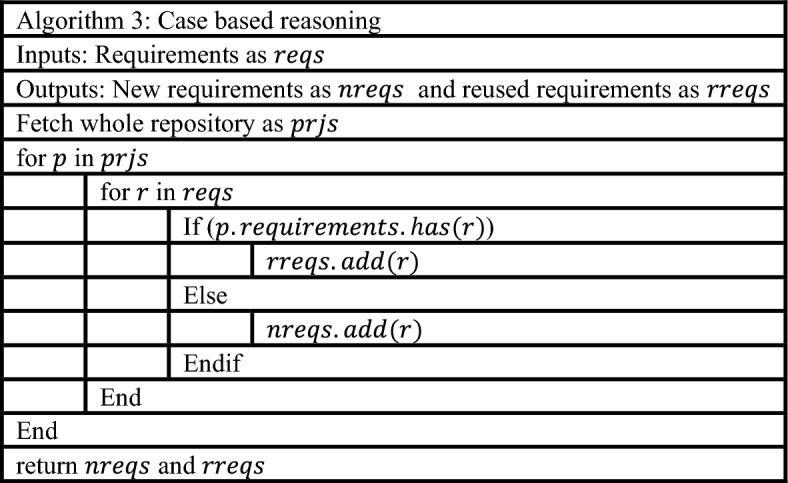
Use of case based reasoning.

### Ethical approval

This study did not include any animal or human participants, nor did it take place in any private or protected sites. For the corresponding places, no special permits were necessary.

## Results and discussion

In summary, this section encapsulates the culmination of our efforts, blending empirical evidence with theoretical insights to construct a comprehensive understanding of the subject matter. Through methodical analysis and thoughtful interpretation, we have strived to unravel the intricacies embedded within the data, yielding results that contribute substantively to the scholarly discourse and chart a course for future investigations.

### Controlled experiment

A controlled experiment was conducted to determine whether the proposed framework can provide the services that it claims. A group of project participants were involved in the framework's execution to deal with requirement changes in the globally scattered Techflow organization. Table 4 categorizes and describes these members.

**Table 4:** Project member’s details.

No.	Project members	No. of participant
1	Clients/stakeholders	6
2	Project manager	4
3	Developer	3
4	Designer	2
5	Change manager	2
6	Quality assurance	2
7	Researcher	7

The proposed framework as well as the existing procedures have been fully implemented. Each of these methods should be used in a comparable setting to allow for a fair comparison. A collection of change requests for project members has been created, including new requirement requests, update requests, and deletion requests.

The project participants were divided into two groups (Group 1 and Group 2). Group 1 used our recommended elicitation technique, which covered communication, coordination, and management challenges, as well as case-based reasoning (CBR) to deal with requirement changes. In the event that stakeholders, such as developers and designers, have questions regarding our ideas, Group-2 employs the traditional technique of project change management.

### Framework execution approach

Despite substantial expertise in the requirement elicitation process, members of Group 1 were unfamiliar with the suggested framework. They directed the members to use the CBR method. All members were involved in the implementation of this newly planned structure.

In a highly dispersed software development environment, the framework provides a very basic and clear design for carrying out this crucial activity of demand modification. The request for requirements elicitation was launched, then forwarded to the project manager, who utilized the CBR technique to develop and attach a report, which was reviewed by all members before a decision was taken. Researchers had already tried with incredibly low settings. But we've all worked together.

### Traditional existing approach

All members of Group 2 were present for the implementation of the typical existing plan. The previous model used many sites with different logins and individuals who did not have access to the entire software development cycle. Because some locations do not have overlapping working hours, extensive meetings are not possible even if there is overlapping time.

This popular modern strategy also limits the use of English. This may result in additional costs, effort, and time, as well as ambiguity, incompleteness, and other challenges such as linguistic and cultural disparities.

Following the implementation of techniques, we concluded that our planned effort was simple to comprehend and achievable, and we strengthened communication, coordination, and monitoring among team members or between the group and stakeholders during the development process.

Our proposed framework, according to our hypothesis, would handle communication, coordination, and monitoring difficulties between consumers and the GSD development team, as well as between the GSD software development team.

The assessment factors based on literature are based on the problems and difficulties of the approaches employed in the RE and GSD methodologies, such as communication, coordination, management, completeness, and correctness, among other things.

In addition, we feel that our proposed strategy outperforms the current conventional technique. As a result, we interviewed people from both groups to investigate some of the variables based on the numerous features described and defined in Table 5.

**Table 5:** Review analysis.

No.	Parameters	PID	Strongly agree	Agree	Neutral	Disagree	Strongly disagree
1	Change management	CM	52	36	20	5	2
2	Communication issues	CI	50	39	8	7	5
3	Task allocation	TA	48	40	16	11	0
4	Collaboration	CO	67	21	15	5	1
5	Reusability	REU	70	24	15	1	0
6	User friendly	UF	60	29	20	0	0
7	Project management	PM	54	32	24	1	0
8	Method adopted	MA	60	30	18	1	0
9	Cost improved	CI	50	35	23	1	0
10	Performance	PER	62	28	21	0	0
11	Effectiveness	EFF	55	30	23	1	0
12	Quality	QU	67	25	17	0	0
13	Time saving	TS	80	15	14	0	0
14	Cultural barriers	CB	52	25	22	7	3
15	Role of expert	ROE	48	25	22	9	5
16	Use of CBR	UOC	60	20	15	12	2
17	Easy to use	ET	60	15	12	12	7
18	Easy to understand	ETU	60	17	18	14	0
19	Division of stakeholders	DS	48	19	20	20	0
20	Classification of requirements	CR	45	20	22	19	2

To comprehend the significance of the distinctions between proposed and existing approaches, we employed reliability analysis to confirm the consistency of the data and component analysis to seek out variance in unobserved latent variables.

**Figure 9:**
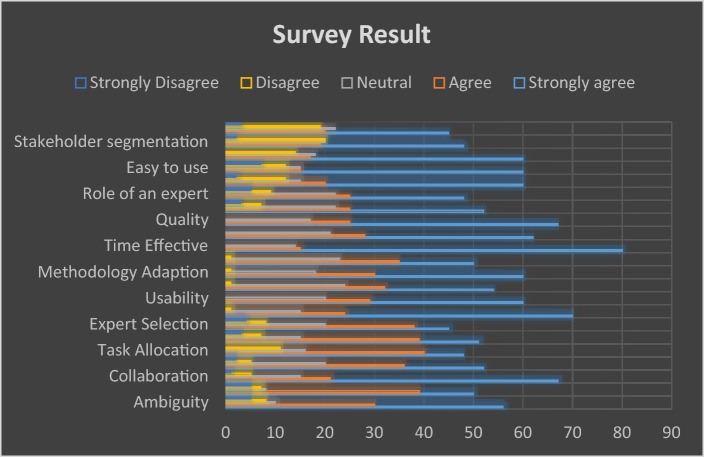
Survey result.

Figure 9 demonstrates that the outcomes are consistent.

We incorporated all criteria from our study into our general poll results, which are highly disagree, disagree, neutral, agree, and severely disagree. Improvements in project management communication issues, expert roles, cultural differences, and requirement classification, as well as other common elements that make our approach the most cost-effective, occurred in large numbers.

### Industrial design

We chose TechFlow's company for the exploratory study and conducted it in a GSD setting because of a copyright issue. The group is focusing on solutions for data management and digital patient care reporting, e-learning, increasing people's independence, urgent delivery, and optimizing your company's operations, among other things. We chose Project A form a broad list of ideas. Project A is the development of web-based and mobile-enabled customer care solutions to provide user-friendly features at a cheaper cost and with enhanced customer satisfaction.

TechFlow has developed a variety of tools and solutions to help GSD overcome communication, collaboration, and leadership difficulties. They chose to adopt our development technique in order to achieve client happiness and create an interactive interface.

We offer the controlled experiment findings in this section and explore how they relate to our study hypothesis.

### Data collection

We began by designing a comprehensive questionnaire that covered relevant factors and distributed it to two distinct companies. The survey process involved meticulous data collection, analysis, and interpretation, yielding unpublished insights into various aspects of their operations and experiences. The data collection process included crafting a detailed questionnaire distributed to Techflow International Company employees. They actively participated, providing thoughtful responses that reflect their experiences and opinions. The collected feedback offers a holistic view of the company's strengths, areas for improvement, and growth opportunities. Key parameters derived from literature guided the formulation of specific questions in the questionnaire. Using an online platform, we efficiently distributed the survey link to target respondents, ensuring structured data collection for valuable insights. As TechFlow navigates the complex landscape of contemporary business, their adept utilization of online communication tools not only catalyzes operational efficiency but also underscores their forward-looking approach. This approach, rooted in harnessing technology for connectivity, positions TechFlow as a model of modern enterprise, where the frontiers of innovation are extended through a harmonious interplay of diverse minds, enabled by the digital tools of the twenty-first century.

We collected information from a study of the literature that includes 10 of the 12 studies, and we have appropriately recorded this data. In order to evaluate various components of the procedure, global software development commonly employs the following types of metrics and performance indicators: Cost, quality, teamwork and communication, geographic location and time zone, results customer satisfaction, teamwork, and security.

### Data analysis

Factor analysis is a statistical approach used to determine whether observable variables have substantial relationships with one another, indicating their grouping within our study framework. This analytical method aids in identifying essential success determinants, comprehending collaborative dynamics, assessing cultural effects, evaluating tool efficacy, and enhancing project management practices within the research environment.

Reliability analysis is a useful method for assessing the overall efficiency of a set of observable variables. It is critical to analyze the stability and trustworthiness of many aspects of the software development process in the context of global software development. We use reliability analysis in our research to assess the consistency and dependability of critical aspects like code reliability, testing and quality assurance procedures, communication protocols, resource allocation strategies, and performance metrics, all of which are critical to the success and efficiency of global software development initiatives.

### Data hypothesis

Using the following research hypothesis, we conducted an empirical study to assess the efficacy of the proposed methodology:

- **Ho1:** How effective is our proposed method for closing the communication gap between clients, stakeholders, and the GSD development team?
  We are aware of communication and coordination gaps between stakeholders and clients in GSD. As a result, the goal of this study is to determine whether our proposed technique improves their communication and coordination.
- **Ho2:** Is it possible that following our recommended technique will greatly reduce the distances between GSD's software development teams?
  We also know that the software development team is spread inside the GSD environment, which creates communication, coordination, and management challenges for team members. As a result, we tested if our proposed technique encourages collaboration and communication among them in this study hypothesis.
- **Ho3:** Could employing our proposed technique increase GSD's requirement for elicitation?
  We evaluated this premise by seeing if our proposed technique for requirement elicitation helps the GSD process.
- **Ho4:** Does our proposed strategy outperform other existing methods for eliciting demand in GSD?

This research hypothesis examines if our proposed technique may better handle requirement elicitation in GSD than other standard elicitation techniques currently in use.

### Organizational context

TechFlow's operations are intricately intertwined with our research focus, leveraging the strengths of a formidable workforce of 670 employees. This includes collaborative teams composed of individuals hailing from diverse backgrounds, with representation spanning Europe and Nigeria. Like any organization, TechFlow's organizational structure serves as the basic blueprint that painstakingly details the orchestration, coordination, and supervision of activities and tasks. Roles and duties are carefully specified within this framework, establishing clear interactions between persons and units. It serves as a guiding framework, defining not only the flow of information but also the decision-making processes and the equal division of tasks. The degrees of power and duty are clearly defined in this structure. TechFlow utilizes an array of online communication tools to facilitate seamless interactions and collaboration among its team members. What distinguishes TechFlow is its proclivity for flexibility within its organizational framework. This versatility enables the organization to respond quickly to the dynamic needs of constantly changing settings, assuring its capacity to prosper in a dynamically growing industry.

### Researcher–organization relationship

In our research endeavors, we have closely aligned ourselves with TechFlow International Technology, a company that bears relevance to our investigative work. While we have not been directly employed by this organization nor established personal interactions.

### Researcher–organization interactions

We initiated the implementation of this model within the organization without any prior connection. We obtained permission from all relevant parties, and their research department conducted the initial review. Subsequently, our work proceeded as planned.

### Research cycles

We assigned particular time limitations to a number of crucial tasks during our study cycle. The first month was devoted to gathering pertinent data, the second month was spent carefully choosing participants, and the third month was utilized to arrange and classify these people according to relevant characteristics. This precise time management allowed us to conduct a thorough and organized study.

Throughout these research cycles, effective communication and collaboration among global teams, as well as the use of appropriate tools and methodologies, are crucial to the success of the global software development project.

### Study duration

Our study lasted two years, with the first year dedicated to doing the research. Following that, we engaged in a rigorous testing phase using the obtained data, employing multiple analysis approaches and methodologies to derive relevant insights over a six-month period.

### Intervention determination

Yes, gaining a deeper understanding of the roles played by development teams, management, and other stakeholders in the design of the proposed algorithmic models would indeed be beneficial for a comprehensive and effective approach to the project. Our approach satisfies the needs of the organization and the researchers through its use of the process. The organization's function significantly shapes the technique and its contributions. Within the organization, we have put practical elements into practice and gathered information from both viewpoints.

### Learned lessons

We must use block chain technology and crowdsourcing in the future as part of our global software development procedures. The capabilities of our project will be improved, and new levels of security and collaboration will be incorporated.

### Parameters comparison

For parameter comparison, we divided the processes into three tables: framework, stakeholders, and client and development sides. The researchers had only experimented with a few settings. However, in our proposed technique, we all worked together, on all components, and combined them, and our results were far superior to previous parameters published in other study studies., as shown in Tables 6, 7, and 8.

**Table 6:** Framework table.

Ref	UOC	ETU	ET	QU	PER	REU	TS	CI	EFF
^1^	X	X	✓	✓	✓	X	X	X	X
^2^	X	X	X	✓	X	X	X	✓	X
^2^	X	X	X	✓	X	X	X	✓	X
^3^	X	X	X	✓	X	X	X	✓	X
^4^	X	X	X	✓	X	X	X	✓	X
^5^	X	X	X	X	X	X	X	✓	X
^6^	X	X	X	X	X	X	X	✓	X
^7^	X	X	X	X	X	X	X	✓	X
^8^	X	X	X	✓	✓	X	X	✓	X
PA	✓	✓	✓	✓	✓	✓	✓	✓	✓

**Table 7:** Stakeholders table.

Ref	COI	CO	CB
^1^	✓	✓	✓
^2^	✓	✓	X
^2^	✓	✓	X
^3^	✓	✓	X
^4^	✓	✓	X
^5^	✓	✓	X
^6^	✓	✓	X
^7^	✓	✓	X
^8^	✓	✓	X
PA	✓	✓	✓

**Table 8:** Client and development side.

Ref	CM	TA	ROE	PM	DS	CR
^1^	✓	X	✓	✓	X	X
^2^	✓	X	X	X	✓	X
^2^	✓	X	X	X	✓	X
^3^	✓	X	X	✓	✓	X
^4^	✓	X	X	✓	X	X
^5^	X	X	X	X	X	X
^6^	X	X	X	X	X	X
^7^	X	X	X	X	X	X
^8^	✓	X	X	X	X	X
PA	✓	✓	✓	✓	✓	✓

### Approaches comparison

**Figure 10:**
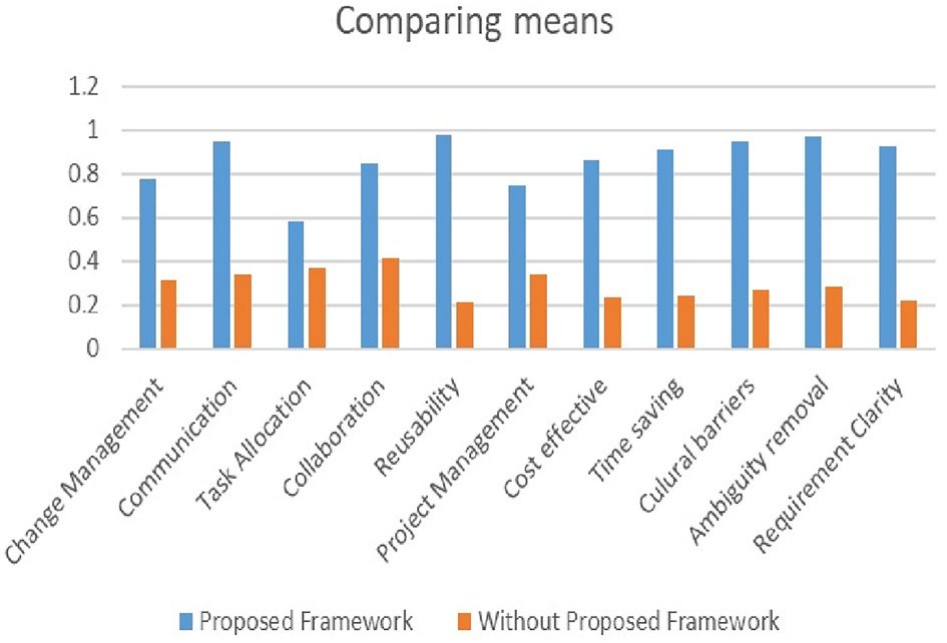
Approaches comparison.

In the approaches comparison we take two frameworks one is previous and second our framework which in Fig. 10.

### Researcher biases

The author and supervisor don’t have any blood relations with the selected organization, named "TechFlow International Technology". The organization was chosen because of its intricate link with our research focus, and research results are gathered after executing our model by the company's concerned team.

## Conclusion and future direction

In past work, various obstacles were encountered during software development, and all work and methods were not combined, resulting in a costly and time-consuming process as well as a disruption in software quality. In our framework, we have a panel of experts, and our prototype includes a virtual project manager as well as several types of developers and designers. They decide on their own job, relieving the project manager of the responsibility. They also involve task allocation, training, extra time and cost savings, enhancing software quality, and decreasing requirement duplication and redundancy. Even requirements that are already in the repository can be reused, and the use of CBR, which comprises different sorts of situations, is essential. We increased framework reusability, which was extremely cost-effective. In the future, we will increase trace interconnectivity while eliciting demands in GSD during the requirement engineering process, as well as their impact on requirement engineering activities. This study will also contribute to the improvement of the requirements elicitation process in cloud and hybrid cloud systems.

## Data Availability

The authors affirm that the data gathered or analyzed, as well as the material supporting the study's findings, are included in the publication.
